# From neuroinflammation to gliomagenesis: immune drivers of malignant transformation in the CNS

**DOI:** 10.3389/fimmu.2025.1682030

**Published:** 2025-12-01

**Authors:** Yingshi Bao, Zixuan Chen, Yutong Su, Tingting Guo, Huaping Du, Xianjun Jia

**Affiliations:** 1 Department of Neurology, Suzhou Ninth People’s Hospital, Soochow University, Suzhou, Jiangsu, China; 2 Department of Neurology, Qidong People’s Hospital, Qidong Liver Cancer Institute, Affiliated Qidong Hospital of Nantong University, Nantong, China; 3 The Second Clinical Medical College, Nanjing Medical University, Nanjing, Jiangsu, China

**Keywords:** gliomagenesis, neuroinflammation, microglia, immunosuppression, STAT3 signaling, glioma-initiating cells

## Abstract

Chronic neuroinflammation is increasingly recognized not merely as a consequence of CNS pathology but as a driver of glioma initiation. Sustained immune activation, induced by trauma, infection, or neurodegeneration, reshapes the brain’s immune milieu in ways that favor malignant transformation. Persistent inflammation activates glial cells, triggers cytokine release, and disrupts the blood-brain barrier, permitting immune infiltration and dysfunction. These changes promote the accumulation and reprogramming of immunosuppressive populations, including regulatory T cells and myeloid-derived suppressor cells, while resident microglia and astrocytes adopt tumor-supportive phenotypes. We highlight signaling axes such as IL-6/STAT3, NF-κB, and TGF-β that connect immune dysregulation to epigenetic instability and the emergence of glioma-initiating cells. By tracing the progression from inflammation to tumorigenesis, we identify opportunities for early immune-based intervention, particularly in individuals with chronic neuroinflammatory conditions.

## 1 Introduction

For decades, the central nervous system (CNS) was viewed as immune-privileged, shielded by the blood–brain barrier (BBB), lacking conventional lymphatic drainage, and sparsely populated with professional antigen-presenting cells. This led to the belief that it was largely isolated from systemic immunity. This view has evolved. The CNS possesses a tightly regulated, responsive immune environment coordinated by resident glia, endothelial cells, and, under specific conditions, infiltrating peripheral immune cells (1).

Neuroinflammation, defined as immune activation within the CNS, may be triggered by infection, trauma, autoimmunity, or neurodegeneration. Acutely, it can be protective, facilitating repair and debris clearance. When prolonged or dysregulated, however, it becomes maladaptive, driving oxidative stress, synaptic dysfunction, glial scarring, and long-term microenvironmental damage (2). Recent research increasingly implicates chronic neuroinflammation not only as a consequence of CNS injury but as an active participant in oncogenic processes, particularly in glioma (3, 4).

Gliomas, and in particular glioblastomas, remain among the most aggressive primary brain tumors. Beyond canonical molecular hallmarks, including mutations in IDH1, amplification of EGFR, and loss of PTEN, the tumor immune microenvironment (TIME) critically shapes disease course and therapeutic resistance (5, 6). Chronic inflammation promotes genomic instability, epigenetic reprogramming, and immunosuppressive niches that enable the transformation of glial precursors into tumor-initiating cells (7). Epidemiological and preclinical evidence links chronic neuroinflammatory states with a possible increase in glioma risk; however, findings are heterogeneous and mechanisms remain under investigation.

In this review, we synthesize how chronic neuroinflammation contributes to gliomagenesis through three connected processes: (1) initiation of neuroinflammation and innate immune activation; (2) remodeling of the CNS immune landscape under chronic stress; and (3) immune-driven selection of resistant, tumor-initiating glial clones. We highlight intervention points along this continuum, focusing on early biomarkers and prevention-oriented strategies.

## 2 Neuroinflammatory triggers and immune initiation

Neuroinflammation begins when resident glia or infiltrating leukocytes detect damage-associated molecular patterns (DAMPs) or pathogen-associated molecular patterns (PAMPs) via pattern-recognition receptors (PRRs) expressed on microglia and astrocytes. Toll-like receptors (TLRs), especially TLR2 and TLR4, are prominent: TLR4 recognizes high-mobility group box 1 (HMGB1), a prototypical DAMP from necrotic neurons, which activates NF-κB and upregulates pro-inflammatory genes (8, 9).

In parallel, the nucleotide-binding domain-like receptor protein 3 (NLRP3) inflammasome senses cytosolic danger signals, including extracellular ATP, mitochondrial dysfunction, and misfolded proteins, leading to caspase-1 activation and maturation of IL-1β and IL-18 (10). PRR and inflammasome signaling initiates and sustains CNS immune responses and has been implicated in early gliomagenesis in animal models (11).

Microglia rapidly adopt a classically activated (M1-like) program characterized by iNOS, IL-1β, TNF-α, and IL-6, aiding pathogen clearance (12). However, prolonged activation sustains oxidative DNA damage in neighboring cells, fosters proliferation, and disrupts neuron–glia communication (13).

Astrocytes likewise respond to IL-1β and TNF-α by producing chemokines that increase BBB permeability and recruit monocytes and T cells, while secreting IL-6 and GM-CSF to reinforce glial activation (14). BBB breakdown allows continued influx of peripheral immune cells—CD4^+^/CD8^+^ T cells, NK cells, and myeloid-derived suppressor cells (MDSCs)—which can become exhausted or immunosuppressive (15, 16). IL-6–JAK/STAT3 signaling supports glial survival and expansion of stress-tolerant precursors, whereas TNF-α and IL-1β promote extracellular matrix remodeling, angiogenesis, and excitotoxicity (17).

Key triggers, receptors, cellular responses, and implications are summarized in **Table 1**. Together, these processes define the early phase of neuroinflammation: a complex, self-reinforcing network of glial activation, cytokine signaling, and immune cell recruitment. While initially aimed at preserving CNS integrity, persistent inflammation may generate a molecular and cellular environment primed for neoplastic transformation. Recognizing and intervening during this window of immune dysregulation could hold promise for glioma prevention or early therapeutic targeting.

**Table 1:** Neuroinflammatory triggers and immune initiation in gliomagenesis.

Category	Key components/processes	Mechanisms & effects	Implications
Triggers	DAMPs; PAMPs; extracellular ATP; protein aggregates (amyloid)	Activate PRRs (TLRs, NLRP3); induce NF-κB and pro-inflammatory genes	Sustained activation → chronic inflammation; early tumorigenesis in models
Receptors	TLR2/TLR4; NLRP3 inflammasome	TLR4–HMGB1 → NF-κB; NLRP3 → caspase-1 cleavage of pro-IL−1β/IL−18	Cytokine release (IL−1β, IL−18); cascade amplification
Glial responses	Microglia (M1-like: iNOS, IL−1β, TNF−α, IL−6); Astrocytes (CCL2, CXCL10, IL−6, GM−CSF)	Acute: clearance; Chronic: oxidative DNA damage, disrupted neuron–glia signaling	Pro-tumorigenic milieu; peripheral immune recruitment
Peripheral recruitment	Monocytes → TAMs; CD4^+^/CD8^+^ T cells; NK cells; MDSCs	BBB disruption; T-cell exhaustion; MDSC expansion	Immunosuppression; impaired cytotoxicity
Pathways	IL−6→JAK/STAT3; TNF−α/IL−1β; GM−CSF	Glial survival; matrix remodeling; angiogenesis	STAT3-linked precursor expansion; pro-growth signaling

## 3 Remodeling of the CNS immune microenvironment

Persistent neuroinflammation progressively remodels the CNS immune milieu into a permissive niche for tumor initiation, characterized by expansion of glioma-associated microglia/macrophages (GAMs), intensified astrocyte–immune crosstalk, and metabolic/checkpoint reprogramming. Below, we detail these processes.

### 3.1 Polarization of microglia and recruitment of macrophages

Microglia, the resident macrophages of the CNS, shift from a pro-inflammatory M1-like to an immunosuppressive M2-like state under chronic stimulation, secreting IL−10, TGF−β, growth factors (EGF, VEGF), and matrix metalloproteinases (MMPs) that promote tissue remodeling, angiogenesis, and suppression of cytotoxic immunity (18). Glioma-derived CSF1 and GM−CSF further regulate this transition (19, 20).

BBB disruption enables peripheral monocytes to infiltrate and differentiate into macrophages (21). Together with resident microglia, they form GAMs, which can constitute a substantial fraction of glioblastoma mass and exhibit transcriptional programs such as CD163, CD204, TREM2, ARG1 conferring immunotolerant, tumor−promoting functions (22).

### 3.2 Astrocyte–immune crosstalk

Astrocytes also play an important role in the process of immune remodeling, mainly through cytokine secretion and direct interaction with tumor cells and immune cells. In the chronic inflammatory environment, astrocytes are in a reactive state and upregulate STAT3 signaling, linked to immunosuppression via PD-L1 expression and CCL2-mediated Treg recruitment (23, 24). Through gap junctions, astrocytes can deliver glutathione and metabolic intermediates to adjacent glioma cells, enhancing oxidative-stress resistance (25). Context-dependent astrocyte states include neurotoxic A1 and tumor-supportive phenotypes secreting lipocalin-2, complement (C3), and MMPs, thereby promoting invasion and immune escape (15).

### 3.3 Neural regulation of immunity in the TME

**Figure 1:**
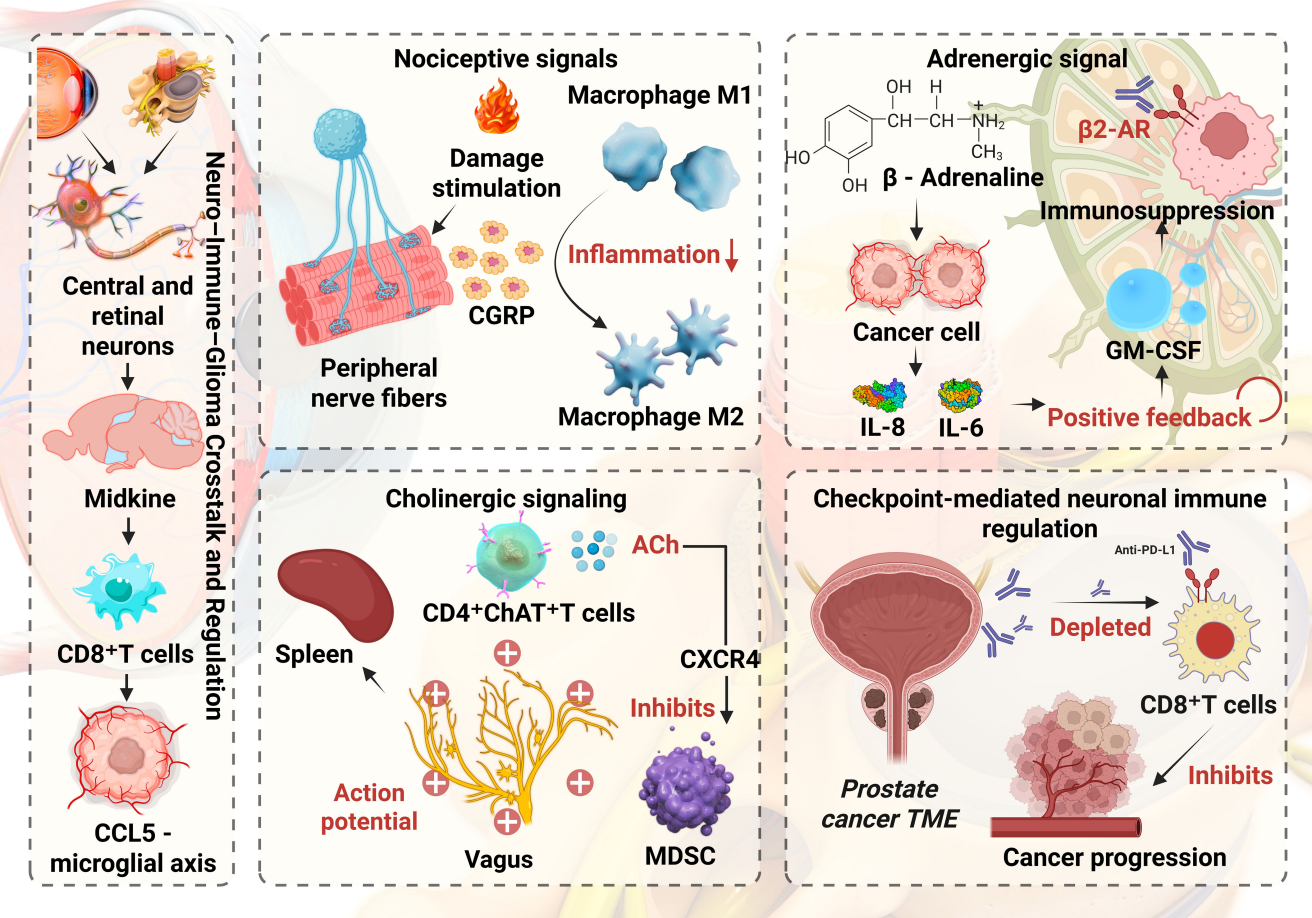
Neuro–Immune–Glioma crosstalk and regulation.

Glioma associated immune cells, including microglia and macrophages, MDSCs and Tregs, are dynamically shaped by nociceptive, adrenergic, cholinergic and checkpoint mediated neuroregulatory pathways. Following tissue injury, peripheral nociceptive fibers release substance P and CGRP. Substance P acting through NK1R supports the survival of activated T cells, enhances proinflammatory cytokine production by macrophages and amplifies CCL5 driven neutrophil chemotaxis and migration. In contrast, CGRP drives macrophages toward an anti inflammatory M2 phenotype, inhibits NLRP3 inflammasome activation and IL 1β release in M1 macrophages and reduces cytokine secretion from macrophages and dendritic cells, thereby dampening antigen presentation and effector T cell activation. β-adrenergic signaling in tumor and myeloid cells induces immunomodulatory cytokines (IL-8, IL-6) and suppressive programs in macrophages while enhancing fatty-acid oxidation in MDSCs and impairing glycolytic reprogramming in CD8^+^ T cells (26–28). Cholinergic vagal signaling activates spleen resident memory CD4^+^ ChAT^+^ T cells, which release acetylcholine to modulate nicotinic receptors on macrophages and secrete trefoil factor 2 that restrains MDSC proliferation through CXCR4 signaling (29). In addition, nerve fibers in the prostate tumor microenvironment can express immune checkpoint molecules such as PD L1 that bind PD 1 on CD8^+^ T cells, promoting their depletion and weakening antitumor immunity (30). **Figure 1** schematically summarizes neuro–immune–glioma crosstalk. Where observations are largely derived from established tumors, we explicitly note uncertainties about their timing relative to pre-tumor states.

### 3.4 Immunosuppressive cell populations and metabolic reprogramming

Regulatory T cells (Tregs) accumulate in chronically inflamed CNS tissue and glioma, driven by chemokine axes such as CCL22–CCR4, CCL2–CCR2, and CXCL12–CXCR4 (31). Beyond increased numbers, Tregs exhibit functional specialization: effector Tregs expressing high CTLA-4 and TIGIT suppress dendritic-cell co-stimulation, while ICOS^+^ Tregs preferentially expand in IL-10–rich niches. Astrocyte and tumor-derived TGF-β stabilizes FOXP3 and enforces suppressive transcriptional programs. In pre-neoplastic settings, microglia-derived IL-10 and CSF1 may precondition perivascular niches to favor Treg retention, blunting early anti-tumor surveillance (32).

Myeloid-derived suppressor cells (MDSCs) encompass polymorphonuclear (PMN-) and monocytic (M-) subsets. PMN-MDSCs release reactive oxygen species and peroxynitrite, nitrating the T-cell receptor and diminishing antigen sensitivity. M-MDSCs deploy ARG1 and iNOS to deplete L-arginine and generate nitric oxide, inducing T-cell cycle arrest (33). Hypoxia and lactate stabilize HIF-1α and drive a ‘metabolic trap’—upregulating CD39/CD73 to convert ATP to adenosine, which signals via A2A receptors on T cells to inhibit cytotoxicity. This adenosinergic circuit emerges early during chronic inflammation when ATP release and ectonucleotidase expression are heightened (34).

Metabolic reprogramming compounds cellular suppression. Glioma and reactive glia increase glycolysis and glutaminolysis, acidifying the interstitium and impairing T-cell receptor signaling. IDO1-mediated tryptophan catabolism accumulates kynurenine, activating the aryl hydrocarbon receptor (AhR) in T cells and macrophages to reinforce tolerogenic states (35). Lipid uptake and oxidation in GAMs and MDSCs, potentiated by CPT1A, supports an M2-like phenotype and limits antigen presentation (36). NAD^+^/SIRT signaling and redox buffering (glutathione, thioredoxin) enable GICs and GAMs to withstand oxidative stress generated during chronic inflammation (37). Collectively, these cellular and metabolic programs not only sustain immunosuppression in established tumors but plausibly predate histological transformation, constituting a permissive niche for malignant initiation.

### 3.5 Recent advances in TAM reprogramming

Therapeutic re-education of tumor-associated macrophages (TAMs) aims to convert immunosuppressive M2-like states into inflammatory, antitumor phenotypes. CSF1R blockade reduces TAM survival and can shift transcriptional programs; however, compensatory recruitment and phenotypic plasticity limit durability. Combinatorial approaches with radiotherapy or oncolytic agents may enhance efficacy by providing danger signals that sustain M1-like activation (38).

Innate agonists, including TLR7/8 ligands, STING agonists, and CD40 agonists, promote antigen presentation and pro-inflammatory cytokine release. Localized delivery is critical to avoid systemic toxicity while overcoming the BBB. Brain-targeting nanoparticles that engage transferrin receptors or utilize RVG29/angiopep-2 peptides can ferry cargo across the BBB, co-delivering cytotoxics and immune adjuvants to both tumor cells and TAMs, thereby coordinating direct tumor kill with myeloid reprogramming (39, 40).

Phagocytosis checkpoints provide complementary targets. Antibodies against CD47 and SIRPα decouple inhibitory cues, permitting opsonization-dependent clearance by microglia/macrophages (41). Microglial receptors such as P2RY12 and TREM2 integrate purinergic and damage signals; modulating these pathways may augment antigen handling and crosstalk with dendritic cells for more effective T-cell priming (42, 43).

## 4 Immune-driven selection and tumorigenic transition

Chronic inflammation does not simply suppress immunity—it imposes selective pressures that favor glial clones equipped to evade or co-opt immune control. Over time, these pressures edit the antigenic landscape, reshape DNA repair and epigenetic programs, and culminate in the expansion of glioma-initiating cells (GICs).

### 4.1 Oncogenic signaling under immune pressure

IL-6/STAT3 and NF-κB function as central hubs that link inflammatory cues to survival, proliferation, and stemness. Sustained STAT3 signaling upregulates anti-apoptotic BCL2 family members and cyclins, while cooperating with HIF-1α under hypoxia to drive angiogenic programs (VEGF) (44). NF-κB activation downstream of TLRs and cytokine receptors induces matrix-remodeling enzymes (MMP2/9), chemokines, and immune checkpoints. These pathways collectively endow pre-malignant glial cells with resistance to stress and immune-mediated killing.

TGF-β can further promote the development of malignant evolution of glial cells by inducing epithelial mesenchymal transition (EMT)-like changes, conferring greater migratory capacity and resistance to immune-mediated killing (45). TGF-β can also downregulate the expression of MHC class I molecules and reduce the “visibility” of these cells to cytotoxic T lymphocytes (CTLs) (46).

### 4.2 Epigenetic alterations and DNA damage

Reactive oxygen and nitrogen species generated during chronic inflammation produce 8-oxoG lesions, single- and double-strand breaks, and replication stress. Activation of ATM/ATR pathways selects for clones with enhanced DNA damage responses or tolerance. APOBEC family cytidine deaminases, induced by inflammatory signaling, can accelerate mutational processes and genomic diversification (47). Inflammation reshapes chromatin landscapes. STAT3 and NF-κB recruit histone modifiers and DNA methyltransferases to promoter and enhancer regions, silencing tumor suppressors or antigen presentation machinery. Microglial cytokines and lactate influence histone acetylation in neighboring glia, promoting dedifferentiation traits associated with GIC phenotypes (48).

### 4.3 Immune editing and antigen presentation

Immune surveillance initially eliminates highly immunogenic clones. As selection proceeds, surviving populations reduce antigenicity or increase inhibitory ligand expression. PD-L1, galectin-9, and CD276 engage PD-1/TIM-3 receptors on T cells, reinforcing exhaustion. Simultaneously, defects in antigen processing, downregulation of β2-microglobulin, TAP1/2, and HLA molecules, decrease MHC-I presentation (49).

Adenosine-rich, hypoxic niches and persistent IL-10/TGF-β signaling favor an ‘immune silent’ state that resists dendritic-cell activation. Where cGAS–STING senses cytosolic DNA from damaged cells, chronic stimulation can paradoxically induce negative feedback and desensitization, blunting type I interferon responses critical for cross-priming (50).

### 4.4 Expansion and ecology of glioma-initiating cells

Under the pressure of inflammation and immune selectivity, GICs with stem-like characteristics gain survival advantages. GICs usually express nestin, Sox2 and CD44, showing significant resistance to oxidative stress, DNA damage and immune-mediated apoptosis (51). These cells are mostly located in chronic inflammatory areas, and the local microenvironment is rich in TGF-β, IL-6 and hypoxia signals, providing a supporting niche (52).

It is worth noting that these initial cell populations also have the mechanism of active inhibition of local immunity, such as the secretion of galectin-1 and prostaglandin E2, which further enhance the formation of the overall immunosuppressive microenvironment. Thus, immune-driven selection acts as a key filter in the inflamed CNS, promoting clonal expansion and evolution of glial cells and the emergence of malignant phenotypes. This transformation is gradual rather than abrupt, reflecting cumulative immune pressure, metabolic constraints, and molecular adaptations.

## 5 Discussion and future directions

Accumulating evidence indicates that chronic neuroinflammation does more than accompany glioma; it progressively reshapes the central nervous system immune milieu into a niche that permits malignant initiation and supports subsequent progression. Early pattern-recognition events and inflammasome activation trigger cytokine cascades and blood–brain barrier dysfunction, after which glial and myeloid populations adopt immunotolerant states. Microglia and infiltrating macrophages converge toward GAM phenotypes with pro-angiogenic and matrix-remodeling programs, astrocytes strengthen immune evasion through STAT3 activity and chemokine networks, and regulatory T cells together with MDSCs enforce suppression. Metabolic rewiring further entrenches dysfunction through lactate accumulation, adenosine signaling, and nutrient competition. In parallel, inflammatory hubs such as IL-6–STAT3 and NF-κB promote stem-like traits, while defects in antigen processing and upregulation of checkpoint ligands reduce immune visibility. These processes unfold in spatially organized niches and show temporal evolution from acute activation toward stable suppression, providing tractable windows for monitoring and intervention.

Clinical translation remains difficult in GBM because profound myeloid suppression, limited neoantigen load in subsets, and the physical constraints of the blood–brain barrier blunt responses to single-agent checkpoint blockade. A more effective strategy begins with microenvironment reconditioning, then mobilizes adaptive immunity. Upstream cytokine and growth-factor pathways such as IL-6-STAT3, TGF-β, and CSF1R represent rational control points; adenosine A2A and IDO1 constitute metabolic brakes that can be released; epigenetic modulators may restore antigen presentation and improve recognition. Myeloid reprogramming with TLR or STING agonists, CD40 engagement, or phagocytosis-checkpoint inhibition complements these approaches and should be deployed with attention to timing, for example peri-resection or immediately after radiotherapy when antigen release and trafficking are favorable. Regional delivery methods including convection-enhanced delivery, focused ultrasound opening, and intrathecal or intraventricular routes improve exposure while limiting systemic toxicity, and brain-targeted nanoparticles or depot biomaterials can sustain local immune modulation along resection margins or infiltrative tracts.

In recurrent disease, the phase 3 cohort of CheckMate 143 found no overall survival advantage for PD-1 blockade compared with bevacizumab and reported a higher objective response with bevacizumab, underscoring the difficulty of overcoming a suppressive ecosystem with single-agent checkpoint inhibition (53). In newly diagnosed disease, CheckMate 498 in MGMT-unmethylated glioblastoma showed no benefit for radiotherapy plus PD-1 blockade over the temozolomide-based standard, and CheckMate 548 in MGMT-methylated or indeterminate tumors did not improve survival when PD-1 blockade was added to chemoradiation (54, 55). These outcomes align with early cold immune microenvironments, lymphopenia from radiotherapy, steroid exposure, and limited trafficking across the blood–brain barrier that together blunt adaptive responses. Cellular therapies illustrate related constraints. An EGFRvIII-directed CAR-T study demonstrated tumor trafficking yet rapidly revealed antigen loss and adaptive resistance *in situ* (56). IL13Rα2-directed CAR-T achieved striking but transient regression in some patients, which highlights antigen heterogeneity and exhaustion (57). A large randomized trial of the EGFRvIII vaccine rindopepimut failed to improve survival in newly diagnosed disease, reinforcing the limitations of single-antigen strategies in a heterogeneous and suppressive context (58).

Much of the causal chain has been inferred from established tumors, which necessitates prospective cohorts of patients with chronic neuroinflammation, faithful models of pre-neoplastic states, and longitudinal tissue or cerebrospinal fluid sampling to separate cause from consequence. Inter-patient heterogeneity in autonomic tone, neuropeptide signaling, and metabolic programs is expected to shape therapeutic sensitivity and may warrant stage-aware and biomarker-guided protocols. Even with these caveats, a coherent model emerges in which sustained neuroinflammation seeds immunosuppressive ecosystems, selects for immune-evasive glial clones, and licenses gliomagenesis. Clarifying the earliest measurable deviations, aligning combinations to disease stage, and refining regional delivery are the most promising routes toward earlier diagnosis and preventive immunomodulation.
